# Maize Silk Biogenic Nanoceria (CeO_2_NPs) Enhanced Sequential Injection-Chemiluminescence Detection of Ferulic, Sinapic and *p*-Coumaric in Yellow Maize Kernels

**DOI:** 10.3390/plants11070885

**Published:** 2022-03-25

**Authors:** Hesham Farouk Oraby

**Affiliations:** 1 Department of Crop Science, Faculty of Agriculture, Zagazig University, Zagazig 44519, Egypt; heshamoraby@gmail.com; 2 Deanship of Scientific Research, Umm Al-Qura University, Makkah 24381, Saudi Arabia

**Keywords:** *Zea mays*, maize silk, biogenic green synthesis, phenolic compounds, nanoceria (CeO_2_NPs), SIA chemiluminescence

## Abstract

The current study demonstrated the capability of using maize silk as a green, simple, clean, safe, and cost-effective platform for the biosynthesis of cerium oxide (CeO_2_NPs). Several spectroscopic and microscopic analyses were employed to characterize the resulted biogenic nanoceria. When the concentration of the CeO_2_NPs was elevated from 25 to 100 ug mL^−1^, the CeO_2_NPs exhibited strong scavenging potential ranging from 60.21 to 75.11% and 56 to 77% for 1,1-diphenyl-2- picrylhydrazyl (DPPH•) and 2-2′-azino-bis(3-ethyl benzothiazoline-6-sulphonic acid) (ABTS) tests, respectively. The quantitative determination of ferulic, sinapic, and *p*-coumaric acids was carried out using an eco-friendly, cost-effective, and optimized ultrasensitive nanoceria enhanced sequential injection-chemiluminescence (SIA-CL) system. The highest amount was presented by the ferulic acid (1636 ± 2.61 ug/g_dw_), followed by *p*-coumaric acid (206 ± 1.12 ug/g_dw_) and sinapic acid (123 ± 2.15 ug/g_dw_). The intrinsic capabilities of the biogenic CeO_2_NPs in enhancing the developed system reveal its potential role in detecting phenolic compounds with great sensitivity.

## 1. Introduction

Reactive oxygen species (ROS) are a challenge to all aerophilic species and are chemically reactive by-product molecules and free radicals [1]. Once the cell is exposed to toxic stimuli, the cytotoxicity of the universally formed ROS overcomes endogenic antioxidant protection machinery causing oxidative stress [2]. The abundance of ROS activates the pathway of cell death programs in both human and plant cells caused by protein oxidation and peroxidation, nucleic acid damage, and enzyme inhibition [3]. However, generating low levels of ROS was found to be important in supporting ordinary physiological and metabolic functions, and in stimulating cellular signaling-mediated pathways related to proliferation and differentiation. Since actively scavenging elevated levels of ROS is vital to the existence of the cell, a large number of phenolic compounds offer the antioxidant capability to scavenge and quench free radicals [4].

Phenolic compounds are naturally occurring molecules that result from secondary metabolism in plants. They are extensively dispersed, and from a physiological standpoint, some play a crucial role in the interaction with environment, especially in the defense response to stresses [5]. The phenolic compounds present in various plants donate their distinguishing appearances, in addition to their taste and color. Moreover, they may be connected to the enhancement of human health due to their extensive array of biological applications, such as their antibacterial, anti-inflammatory, anti-allergic, and antioxidant capacities [6]. Phenolics can originate in both free/soluble and bound/insoluble fractions. They have been extensively studied in maize, and it was reported that maize contains high concentrations of those molecules when compared with other cereals [7]. In maize kernels, approximately 98.9% of phenolics exist in the insoluble fraction (ferulic, sinapic, and *p*-coumaric acids). Ferulic acid (FA) (3-methoxy-4-hydroxycinnamic acid) is mostly dominant in the pericarp and aleurone. It is ester-linked to hemicellulose chains (in the arabinoxylan residues) in the plant cell wall and polymerized in lignin structure by ether linkages [8]. *p*-Coumaric acid (CA), with greater presence in the insoluble fraction, is the second most abundant phenolic compound in maize kernel after ferulic acid. In the insoluble fraction, it is largely lignin linked, and slightly embedded in polysaccharides in the cell wall [6]. Sinapic acid (SA), a cinnamic acid derivative, is broadly distributed in different sources of plants such as maize, rye, fruits, and vegetables. Pharmacologically, it has been considered a potent antioxidant and peroxynitrite scavenging factor [9]. Nevertheless, the soluble fraction displays better chemical diversity, which differs based on the grain color [6].

Maize silk (*Stigma maydis*), the lengthened stigma of the maize female flower and a famous Chinese folk herbal medicine, is an abundant global waste byproduct in maize cultivation. It exhibits strong bioactivity effects, such as antioxidant, antidiabetic, anti-obesity, antidepressant, anticancer, and anti-nephrotoxicity effects [10]. Biochemical studies showed that maize silk possesses volatile oils, steroids, saponins, polysaccharides, alkaloids, flavonoids, organic acids, and other phenolic compounds [11].

Nanobiotechnology exploits nanotechnology techniques for developing and improving different biotechnological processes and products. Alternatively, bionanotechnology benefits from natural and biological building blocks, and utilizes biological specificity and activity schemes to develop modern and exceptional nanoscale structures [12]. These technologies are strategic empowering tools that aid the establishment of novel products and help the progression of novel supplies to be applied in diverse disciplines such as medicine, manufacturing, computing, and agriculture sciences [12]. The use of nanoparticles is advantageous in addressing many difficulties in various fields, such as energy, medical diagnostic, computer chips and artificial intelligence, industrial and agriculture applications. Recently, the increasing necessity for more environmentally benign routes in material synthesis has increased the demand for plants and plant-based products to manufacture cost effective, energy proficient, simpler, safer, and non-toxic schemes for metal nanoparticle synthesis as an alternative to traditional synthesis [13].

Cerium, the rare earth element that belongs to the lanthanide series, integrates with oxygen, developing cerium oxide nanoparticles (CeO_2_NPs, nanoceria). The excellent catalytic and antioxidant properties of cerium oxide (CeO_2_) result from the cerium thermodynamic efficiency of redox cycling between Ce^3+^ and Ce^4+^ states [14]. Thus, nanoceria have received a noteworthy focus in different applications, such as pharmacological agents, drug delivery, bio-scaffolding, catalyst converters, fuel additives, and self-regenerating antioxidants. Green bio-directed synthesis of nanoceria with distinctive and tunable characteristics provides more novel keys for antioxidant determination due to chemical stability and increased surface area and alleviates concerns about biocompatibility.

Several plant extracts have been considered as the most abundant, efficient, safe, and rich sources for green synthesis of CeO_2_NPs. Although different plant parts, such as flowers (*Hibiscus sabdariffa*), seeds (*Salvia macrosiphon*), stems (*Euphorbia tirucalli*), peel (*Moringa oleifera*), and shell (walnuts) have been exploited for synthesizing CeO2NPs, most of the studies have employed leaf extracts (*Gloriosa Superba* and *Oleo Europaea*) as reducing and stabilizing agents due to their richness in metabolites [15]. To the best of our knowledge, employing maize silk for green synthesis of CeO_2_NPs has not been reported yet.

For the past several years, a significant quantity of liquid chromatographic (LC) systems have been dedicated to separating and quantifying an enormous variety of active molecules, such as phenolic compounds from plant extracts. However, these systems face various challenges, such as low abundance, limited separation, and matrix interference. Despite the improvement in the liquid chromatography-mass spectrometry (LC-MS) system, which performs better than the frequently utilized systems regarding selectivity and sensitivity, better detection limits and higher selectivity are still needed when analyzing low or trace levels of phenolic compounds [2].

Several chemiluminescence (CL) systems based on flow methodologies have successfully been established. These systems allow a detecting sensor to directly be exposed to a reaction zone. This enhances satisfactory light capability emission from the transient excited state intermediates formed in the CL reactions. Currently, because of its effortlessness, sensitivity, precision, and dependability, particularly at the nanoscale level, sequential injection analysis (SIA) has captured a large amount of attention for the determination of different constituents [2].

Hence, the current study aimed to biosynthesize eco-friendly nanoceria (CeO_2_NPs) using maize silk extract, and to use it to acquire a facile, highly sensitive sequential injection-chemiluminescence (SIA-CL) analysis scheme for nanoscale detection. This scheme was enhanced and employed in determining ferulic, sinapic, and *p*-coumaric acids in the bound fraction of yellow maize kernels.

## 2. Materials and Methods

### 2.1. Chemicals and Reagents

Sigma–Aldrich Chemical Co. (St. Louis, MO, USA) provided Cerium (III) nitrate hexahydrate ([Ce (NO_3_)_3_·6H2O], 99%), cetyltrimethylammonium bromide (CTAB, 98%), ferulic acid, 99%, DPPH• (1,1-diphenyl-2-picrylhydrazyl, 97%), ABTS (2-2′-azino-bis(3-ethylbenzthiazoline-6-sulfonic), ascorbic acid, 99%. Fisher Scientific (Pittsburgh, PA) provided hydrochloric acid (36.5%), ethyl acetate (99.5%), and methanol (99.9%). Approximately 100 mL of 1.0 × 10^−2^ mol L^−1^ sodium hydroxide (NaOH, 98%) (WinLab, East Midlands, UK) was used to prepare a 5-amino-2,3-dihydro-1,4-phthalazinedione (luminol, 97%, Sigma-Aldrich, Hamburg, Germany) stock solution 1.0 × 10^−2^ mol L^−1^. A potassium ferricyanide (K_3_Fe(CN)_6_, 97%,WinLab, East Midlands, UK) 1.0 × 10^−2^ mol L^−1^ solution was prepared by dissolving 0.0329 g in 100 mL of water. In this study, water was ultrapure and there was no need for further purification as analytical grade chemicals were used. All experiments were optimized according to the laboratory conditions.

### 2.2. Plant Materials and Samples

Maize (*Zea mays* L. family Poaceae) silk and kernel samples were obtained from the yellow corn SC167 provided by the Agriculture Research Center, Egypt. After careful washing, the obtained maize silk samples were oven dried for 24 h at 60 °C, then crushed into fine powder, vacuum packed, and stored at −20 °C until further use. The maize silk extract (MSE) was prepared by heating 200 mL of distilled water containing 2 g of maize silk powder at 60 °C for 1 h under constant shaking using a water bath shaker (Memmert, Germany), allowed to cool to room temperature (26 °C ± 1), and filtered using a Whatman filter paper (No. 1). The resulting solution was collected and centrifuged at 4000 rpm for 40 min. MSE, obtained as the supernatant, was stored at 4 °C until further use. Kernels were sun dried and milled, passed through 250 mm mesh, and thoroughly mixed. Samples were stored for 1–3 days at −20 °C until analyzed.

### 2.3. Extraction of Bound Phenolic Compounds and Ferulic, Sinapic, and p-Coumaric Acids

The extraction of bound phenolics was carried out using the improved microscale process adopted by [16] with minor modifications. Briefly, soluble phenolic acids were extracted by adding 7 mL of 80% methanol to 0.5 mg of maize sample and mixed for 5 min with a vortex. With constant agitation, the samples were incubated at 26 °C for 15 min, followed by 10 min centrifugation at 5000 rpm. After discarding the phenolic acids in the free-form, 0.5 g of maize pellet residue was used to extract ester-linked phenolic acids. At 90 °C, alkaline hydrolysis was carried out using 5 mL of 2M NaOH for 1 h. HCl (2M) at pH 2 (5 mL) was used to acidify the extract. Thereafter, *n*-hexane was applied to remove lipids. Three washing rounds with ethyl acetate were applied to recover the phenolic acids. Afterward, ethyl acetate was removed to dryness and samples were resuspended in methanol, filtered, and stored at −20 °C until further analyses. All extraction procedures were performed in triplicate.

Another experiment was carried out to isolate ferulic, sinapic, and *p*-coumaric acids by applying the samples on a “TLC silica gel 60 F254” aluminum sheets stationary phase with chloroform: methanol: water: glacial acetic acid (6:5:1:1, *v*/*v*/*v*/*v*) and chloroform: methanol: glacial acetic acid (7:3:1, *v*/*v*/*v*) as a mobile system regarding different standards to isolate the three selected phenolic compounds [17].

### 2.4. Biogenic Synthesis of CeO_2_NPs (Nanoceria) Using Maize Silk

The biogenic synthesis of nanoceria was conducted by reducing cerium (III) nitrate hexahydrate [Ce (NO_3_)_3_·6H2O] (0.5 g) using 100 mL of MES solution as a reducing agent in the presence of 5.0 mL of 1.0% CTAB (*w*/*v*) as a stabilizing agent. After mixing, the samples were heated for 2.5 h in a water bath under continuous stirring at 250 rpm. The mixture was further ultrasonicated at power of 150 W and frequency of 35 kHz for 12 min to obtain a homogeneous dispersion. A pale-yellow solid precipitate was achieved after cooling to 27 °C. Precipitates were constantly centrifuged at 12,000 rpm for 7 min, and then oven dried overnight at 95 °C to eliminate any additional impurities. The nanoceria concentration was estimated using the equation C_NP_ = T_NP_/T_A_, where T_NP_ equals the number of nanoceria and T_A_ equals Avogadro’s number (6.022 × 1023).

### 2.5. Spectroscopic and Microscopic Characterization of the Prepared Nanoceria

To confirm the formation of the nanoceria, absorption wavelengths ranging from 200 to 500 nm were used to determine the UV-Vis spectrum using a Shimadzu UV-2700 spectrophotometer (Kyoto, Japan). Using Fourier transform infrared spectroscopy (FTIR) analysis (Spectrum BX spectrometer, PerkinElmer Ltd., Yokohama, Japan), the functional groups in maize silk that contributed to the preparation of the nanoceria were detected and measured in the IR region of 4400–400 cm^−1^. The analysis of XRD was conducted to determine the crystalline phase of the biogenic synthesis of CeO_2_NPs using maize silk extract. Scanning Electron Microscopy (SEM, JSM-7610F, JEOL, Massachusetts, USA) along with Energy Dispersive X-ray spectroscopy (EDX) were engaged in the characterization of surface morphology of the biosynthesized particles. The size and stability of the particles were assessed using zeta potential (ZP) and a dynamic light scattering (DLS) analyzer (dynapro1Plate Reader III, Waytt, Japan). The biogenic CeO_2_NPs thermal stability was assessed using a thermogravimetric and differential scanning calorimetry (TGA/DSC) analyzer (Seiko Exstar 6300, Tokyo, Japan) within a range of temperature of 25–650 °C.

### 2.6. SIA-CL Procedure

A FIAlab-3500 instrument, under automatic control, was used in all experiments. A mixture of luminol (60 mL of 1.0 × 10^−4^ mol L^−1^) was added to the CeO_2_NPs (40 mL of 2.4 × 10^−7^ mol L^−1^), 30 mL kernel sample extract, and 60 mL of 1.0×10^−4^ mol L^−1^ potassium ferricyanide. Through the eight-way injection valve, the holding coil received the aspirated mixture at a flow rate of 100 mL s^−1^ which was constantly flushed into the flow-through cell situated anterior to the cell of detection of the photomultiplier tube (PMT). Monitoring the emission of the resulting reaction was performed. Per test solution, the experiment was conducted in triplicate analysis cycles. Calculations were expressed in ng mL^−1^ and converted into ug/g.

### 2.7. The Suggested Control Program

For all calibration measures and the experimental investigations of each sample, the SIA-CL control program was executed. Signal cycles were conducted for 45 s, and 80 h^−1^ was the final sample throughput.

### 2.8. The Plotted Calibration Graphs

Under optimum conditions and at 10 experimental points, calibration curves for determining the three phenolic compounds (ferulic, sinapic, and *p*-coumaric acids) were conducted by plotting the intensity of CL vs. the tested solution concentration. Height peaks were attained after triplicate detections for each aspirated sample. Conventional linear regression was employed for fitting the curves.

### 2.9. Antioxidant Activity

#### 2.9.1. Antioxidant Activity Using (DPPH•) Radical Scavenging Assay

1,1-diphenyl-2-picrylhydrazyl (DPPH•) was used to determine the antioxidant activity according to the protocol of [18]. Different concentrations of MSE and CeO_2_NPs samples (25, 50, and 100 μg mL^−1^) were blended with 100 μM DPPH• ethanolic solution. Reactions were performed in a microplate (96 wells) and dark incubated at 27 °C for 35 min. Optical densities were assessed at 517 nm. For comparisons, the chosen positive control was ascorbic acid. Determination of all curves were performed in quadruplicate. The scavenging activity was recorded using the equation:

% Scavenging activity = [(A_control_ − A_sample_)]/A_control_ × 100

where A_control_ is absorbance for control and A_sample_ is absorbance for samples.

#### 2.9.2. 2-2′-Azino-bis(3-ethylbenzthiazoline-6-sulfonic acid) Radical Cation (ABTS +) Scavenging Activity

The 2-2′-azino-bis(3-ethylbenzothiazoline-6-sulphonic acid) (ABTS) free radical scavenging assay was also employed to test the antioxidant capacity of MSE and CeO_2_NPs utilizing the method adopted by [19]. Briefly, a working solution was prepared by mixing equal amounts of 7.4 × 10^−3^ ABTS and 2.6 × 10^−3^ mol L^−1^ potassium persulfate solutions in the dark for 12 h. Different sample concentrations were mixed with the ABTS working solution (25, 50, 100 μg mL^−1^) for 2 h in the dark. Ascorbic acid was used as a control. Using the UV-Vis BioTek microplate, absorbance was recorded at 518 nm and the following equation was used to calculate the scavenging potential:

% ABTS free radical scavenging potential = [(A_control_ − A_sample_)]/A_control_ × 100

where A_control_ is absorbance for control and A_sample_ is absorbance for samples.

### 2.10. Statistical Analyses

Statistical analyses were performed in replicates using a *t*-test and one-way ANOVA. The values of means and standard deviations were employed to express the data at *p*-value < 0.05.

## 3. Results

### 3.1. Characterization of the Biogenic Synthesized CeO_2_NPs

The biogenic synthesized cerium oxide nanoparticles were characterized using various methods such as UV-Vis, FTIR, XRD, DLS, ZP, SEM, and EDX.

**Figure 1:**
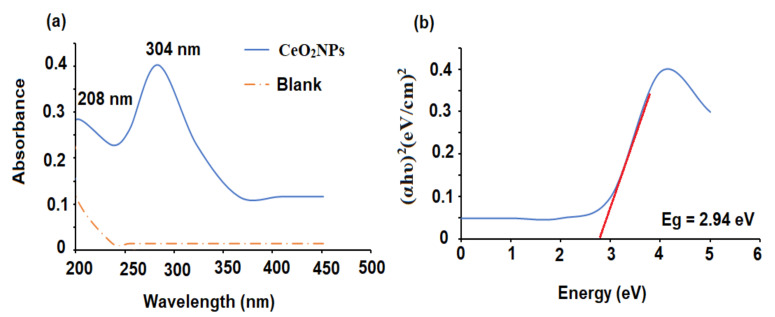
(**a**) UV-Vis spectrum of CeO_2_NPs against deionized water as blank and (**b**) calculated bandgap energy of the biogenic CeO_2_NPs.

The biogenic reduction of CeO_2_NPs was measured spectrophotometrically in the range of 200–500 nm. The absorbance spectrum showed the typical absorption peaks of Ce^3+^ and Ce^4+^ at 208 and 304 nm, respectively (Figure 1a). The appearance of Ce^3+^ on the surface of the nanoparticles is crucial for the antioxidant potential of CeO_2_NPs. The bandgap energy was calculated using the absorption data collected from the UV–Vis spectrophotometer by applying the equation:

Eg = hυ = hc/λ

where h, c, and υ represent the Planck constant, velocity of light, and the wavelength, respectively.

The bandgap of the nanoceria was found to be 2.94 ± 0.01 eV (Figure 1b). The obtained bandgap was lower than the reported bandgap of bulk CeO_2_ (3.2 eV) [20]. It has been well addressed that the reduction in particle size causes an increase in bandgap values as an influence of quantum confinement. The presence of remarkable fractions of Ce atoms (either Ce^3+^ or Ce^4 +^) on the outer surface helps oxygen vacancies and defects to affect bandgap values [21].

**Figure 2:**
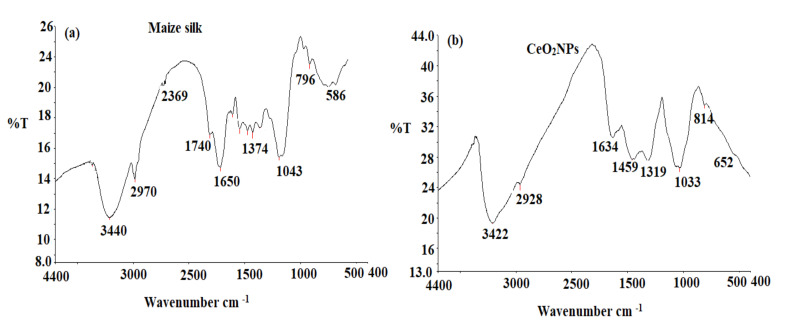
FTIR spectra of (**a**) maize silk extract and (**b**) biogenic CeO_2_NPs detected at 4400–400 cm^−1^.

FTIR spectroscopy was utilized to determine the existence of active functional groups in samples. The FTIR analysis was conducted in the range of 4400–400 cm^−1^. In the FT-IR spectrum of maize silk (Figure 2a), the band at 3440 cm^−1^ existed due to O-H stretching vibrations in water, phenols, and N-H stretching in amino acids. The observed bands at 2970 and 2369 cm^−1^ indicated the existence of both strong N-H stretching of amine compounds and a strong O = C=O carbon dioxide bond, respectively. Different bands were observed at 1740, 1650, 1374, and 1043 cm^−1^ corresponding to a strong C = O stretching aldehyde, a strong C = O stretching o-lactam, a medium C-H bending alkane, and a CO-O-CO strong stretching of anhydride group, respectively. Furthermore, two bands were observed at 796 and 586 cm^−1^ that correspond to medium C = C bending alkene (trisubstituted0 and strong C-I stretching halo compound, respectively. The obtained results confirmed the presence of various functional groups that may be related to the phytochemicals in maize silk extract [22]. The FTIR spectrum of CeO_2_NPs (Figure 2b) showed different absorbance peaks at 3422, 2928, 1634, 1459, 1319, and 1033 cm^−1^, which corresponded to the presence of O-H stretching of water, strong N-H stretching of amine salt, weak C-H bending aromatic compound, medium C-H bending alkane (methyl group), C-N stretching aromatic amine, and strong C-O stretching alkyl aryl ether, respectively. Moreover, two absorption peaks were observed at 814 and 652 cm^−1^ corresponding to medium C = C bending alkene trisubstituted and O-Ce-O nanoparticles, respectively [23].

**Figure 3:**
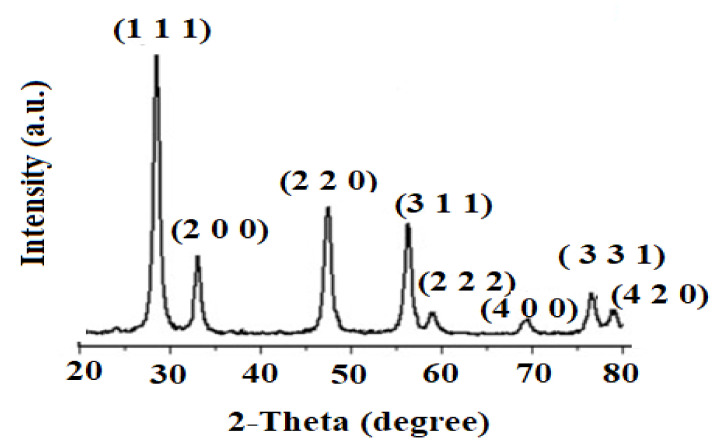
Spectrum of X-ray Diffraction Spectroscopy (XRD) showing the crystal planes corresponding to the crystallite phase CeO_2_NPs.

The analysis of XRD was conducted to determine the crystalline phase of the biogenic synthesis of CeO_2_NPs using maize silk extract. The recorded XRD pattern (Figure 3) was measured at the rate range of (20–80°) 2θ per min. The recorded peaks were found at 28.3° (1 1 1), 32.9° (2 0 0), 47.1° (2 2 0), 56.1° (3 1 1), 58.7° (2 2 2), 69.1° (4 0 0), 76.2° (3 3 1), and 78.7° (4 2 0). The obtained results matched the pattern of standard diffraction of cerium oxide (JCPDS card number 34–0394) and the crystal structure of the CeO_2_NPs samples was determined to be cubic. The identified peaks of carbon and nitrogen were due to the presence of phytochemicals as capping materials on the surface of nanoparticles. The crystallite size was calculated using the Debye–Scherrer [24] equation:

D = 0.89 λ/β Cos θ

where λ (1.54 Å), θ, and β are the X-ray wavelength, angle of Bragg diffraction, and the full width at half maximum, respectively. The estimated crystallite size value was 17.9 ± 1.2 nm.

**Figure 4:**
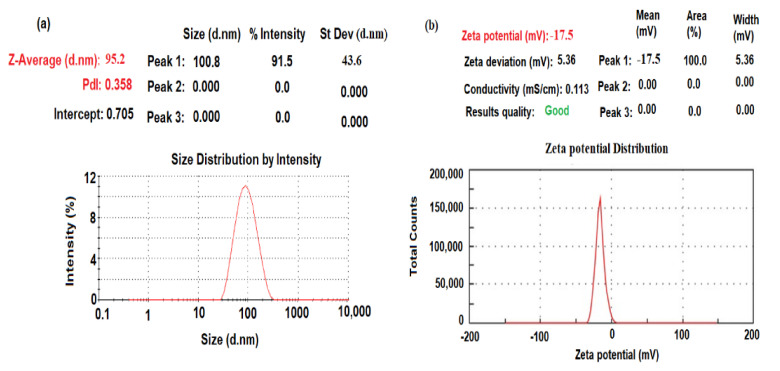
(**a**) DLS and (**b**) zeta potential of biogenic synthesized CeO_2_NPs using maize silk extract.

The dynamic light scattering (DLS) method was applied to measure the mean average size diameter and size distribution due to the intensity of the biogenic synthesized CeO_2_NPs. The particle size distribution was measured using the Zetasizer Ultra particle size analyzer (Malvern Panalytical Ltd., Malvern, UK). As demonstrated in Figure 4a, the particle size distribution of CeO_2_NPs was approximately 95.2 ± 1.2 nm. The size distribution profile exhibited one remarkable peak with 91.5% intensity and a polydispersity index (PdI) equal to 0.358, which suggests that the biogenic synthesized nanoceria had very small agglomeration patterns [25]. The obtained results confirmed that the biogenic synthesized particles using maize silk extract are in the nanoscale form.

The zeta potential of particles with negative values of about −17.2 mV indicated a strong negative charge (Figure 4b). This negative zeta potential suggests that the reduction process can be conducted through the surface-capped plant phytochemicals such as polyphenol compounds present in maize silk extract and adsorbed on the surface of the metal oxide nanoparticles.

**Figure 5:**
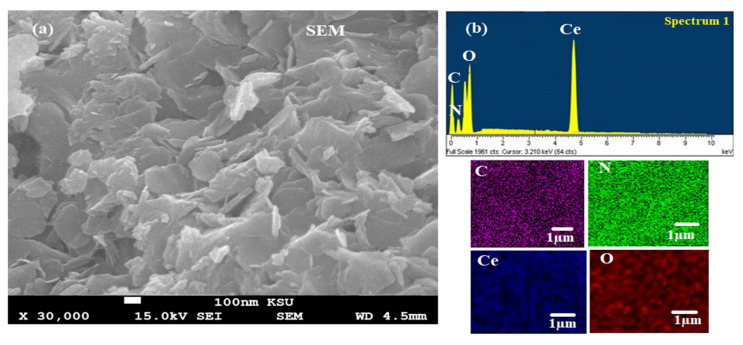
(**a**) SEM of biogenic CeO_2_NPs using maize silk extract and (**b**) EDX of the synthesized CeO2NPs with their elemental mapping.

The morphological shape and particle size of the biogenic CeO_2_NPs were characterized using SEM analysis (Figure 5a). The average size of CeO_2_NPs was in the range of 80–100 nm, confirming the formation of nanoparticles. Typically related to the biogenic nanoparticles, most of the nanoceria were significantly uniform in their dimensions, with slight agglomerations. This can be attributed to the fact that biogenic nanoparticles have a larger surface area and the stable affinity among them causes little aggregation or agglomeration.

The atomic arrangement and chemical content of the CeO_2_NPs was determined by EDX mapping analysis (Figure 5b). The outcomes demonstrated that the weight percentages of Ce, O, C, and N were 75.15%, 19.96%, 3.16, and 1.73%, respectively. The presence of trace amounts of C and N resulted from the presence of phytochemical compounds in maize silk extract that capped the nanoceria surfaces.

**Figure 6:**
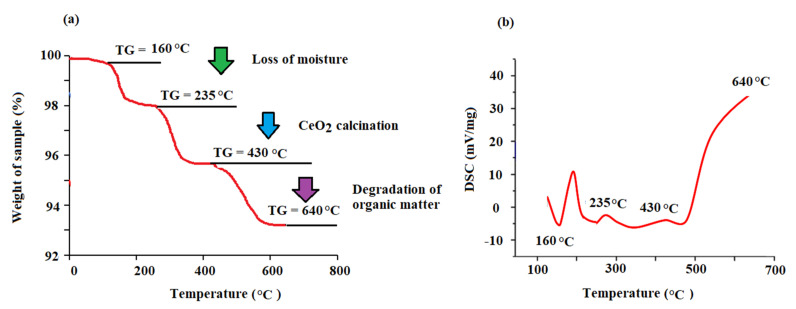
(**a**) Thermogravimetric (TGA) and (**b**) diffraction scanning calorimetry (DSC) of the biogenic CeO_2_NPs.

The TGA/DSC mode was used for the thermal behavior assessment of the biogenic CeO_2_NPs in a temperature range of 25–650 °C (Figure 6). The achieved peaks at different temperatures revealed an overall weight loss of approximately 6.6%, demonstrating substantial thermal stability of the CeO_2_NPs (Figure 6a). Furthermore, the thermogram and diffraction scanning calorimetry (DSC) displayed three distinguished areas of weight loss (Figure 6b). From the curve of TGA, the peaks emerging at 160, 235, and 430 °C were linked to the loss of moisture and volatile components from the surface of particles (2.0% weight loss), full calcination of CeO_2_ (2.2% weight loss), and production of CeO_2_NPs from the complete degradation of organic matters (2.4 w% loss), respectively. After 430 °C, no further loss occurred as no peaks emerged. This proves that the low-temperature calcination of CeO_2_NPs occurred at 430 °C. These results demonstrate the reduction capacity of maize silk bioactive compounds in the establishment of the proper formation of metallic CeO_2_NPs from CeO_2_.

### 3.2. Optimization of CL Conditions

**Figure 7:**
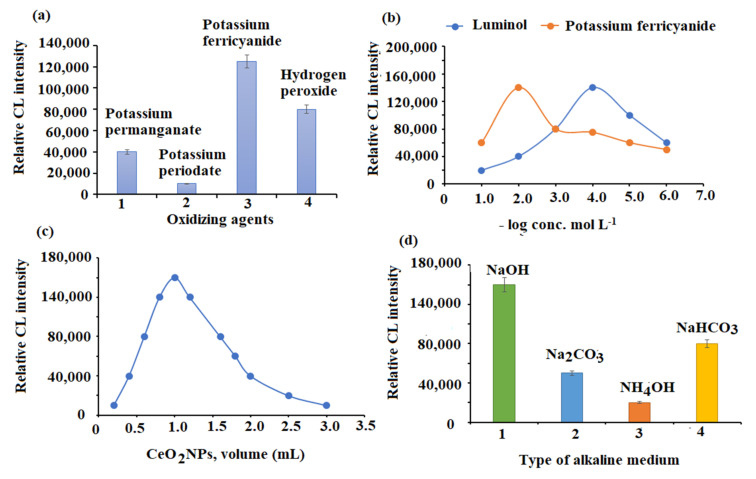
Optimization of SIA conditions: (**a**) selection of suitable oxidizing agent, (**b**) effect of luminol and potassium ferricyanide concentrations, (**c**) effect of CeO_2_NPs volume, and (**d**) selection of suitable alkaline medium.

The choice of an appropriate oxidizing agent is one of the most important parameters that has a significant impact on CL reactions. Hydrogen peroxide, potassium ferricyanide, potassium periodate, and potassium permanganate were studied. Both potassium ferricyanide and hydrogen peroxide revealed strong recorded CL signals. Sharper and stronger CL signals were produced by potassium ferricyanide compared with hydrogen peroxide. Furthermore, a poor CL signal was obtained when potassium permanganate and potassium periodate were employed (Figure 7a). Consequently, the luminol-potassium ferricyanide CL system was chosen for further experiments.

Several concentrations in the range of 1.0 × 10^−5^–1.0 × 10^−1^ mol L^−1^ of potassium ferricyanide and luminol were used to adjust the reagent concentration. A significant elevation in CL intensity was revealed at 1.0 × 10^−2^ and 1.0 × 10^−4^ mol L^−1^ concentrations for both potassium ferricyanide and luminol, respectively (Figure 7b). Since the CeO_2_NPs solution volume can significantly influence the CL intensity of the luminol-ferricyanide system, the CeO_2_NPs volume was tested over the range of 0.5–4.0 mL. CL intensity was sharply increased when using 1.0 mL of CeO_2_NPs (Figure 7c). Therefore, this volume was preferred for the remainder of the study.

The influence of alkaline media was also tested. A sharp CL signal was revealed when a concentration of 1.0 × 10^−2^ mol L^−1^ sodium hydroxide (pH = 10) was added. A significant reduction in CL signal was noticed when ammonium hydroxide, sodium carbonate, and sodium bicarbonate were used (Figure 7d).

The aspirated sample volume and reagents were measured and precisely optimized. It was found that 50, 30, and 30 μL of luminol, CeO_2_NPs, and potassium ferricyanide, respectively, were the optimum aspirated volumes. Moreover, 50 μL was the optimum volume for the tested sample. A minute and half was required to complete flushing through the holding cell between cycles of the analysis. Moreover, when the range of 20–120 μL s^−1^ was used to test the effect of the flow rate on CL intensity, the optimum flow rate was found to be 100 μL s^−1^, and this rate was chosen for the further investigations.

### 3.3. SIA-CL Procedure

Volumes of mixed nanoceria–MSE test solution, luminol, and potassium ferricyanide were optimized. The mixture was automatically coil aspirated, the detector received the mixed zones, and peaks of CL signals were documented. The results showed no significant change in either the oxidation state (chemical properties) or the size (physical properties) of the nanoceria used to catalyze the luminol radical reductions during the reactions of chemiluminescence. This catalysis expresses the exchange between the luminol radicals (unpaired electrons) and the nanoparticles (conduction band electrons) [26].

### 3.4. Method Validation

According to ICH guidelines (ICH Q2B, 1996), several methods of validation were carried out including specificity, linearity, limit of detection (LOD), limit of quantification (LOQ), accuracy, precision, and robustness.

#### 3.4.1. Specificity

During the reactions of chemiluminescence, hydrogen peroxide is disrupted by superoxide produced by the nanoceria-activating luminol ions onto the active surface of particles. Returning to their ground state, these activated ions release illumination. Furthermore, oxygen-containing intermediate radicals can react with various ions, and organic molecules that contain hydroxyl and amino groups. This reduces the CL intensity as a result of changing chemical reactivity, catalytic activity, and redox properties of the luminol mixture [27]. Therefore, the selectivity of the SIA-CL method regarding the detection of the tested phenolic compounds was examined in the existence of several competitive ingredients naturally present in maize kernels (Table 1). These ingredients comprised different minerals, such as Na^+^, K^+^, Fe^3+^, Mn^3+^, Mg^2+^, Cu^2 +^, and Zn^2+^, in addition to some amino acids (lysine and tryptophan) and carbohydrates (starch, sucrose, glucose, and fructose). Moreover, vitamins such as vitamin E, thiamine (vitamin B1), riboflavin (vitamin B2), niacin (vitamin B3), and ascorbic acid were also used to investigate the selectivity of the suggested SIA–luminol–ferricyanide–CeO_2_NPs system. The relative error (less than ±5%) in the CL signal was considered the tolerable limit of the competitive ingredient. The interfering substances did not record any significant influence (*p* ≤ 0.05) on the CL signal of the phenolic compound detection. These results revealed that luminol intensity was not reduced by any of the foreign constituents that could act as molecular traps or quenchers (inter-molecular electronic energy transfer). Furthermore, it is worth noting that luminol CL signals in redox reactions can be enhanced by the high catalyzing properties of Fe^3+^ and Mn^3+^ present in the biological molecules [27].

#### 3.4.2. Linearity

**Figure 8:**
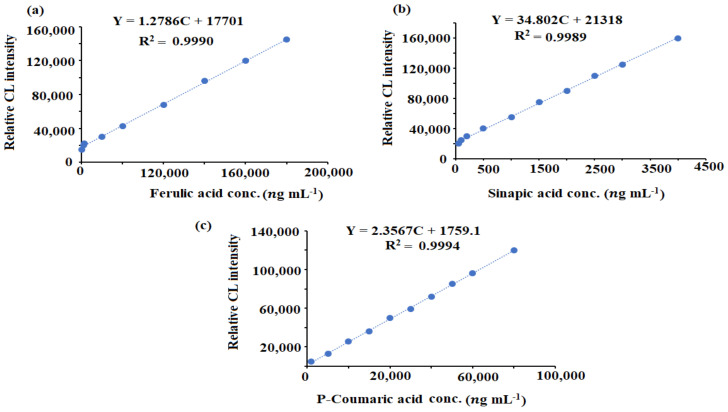
Calibration graphs of (**a**) ferulic acid, (**b**) sinapic acid, and (**c**) *p*-coumaric acid using the luminol–ferricyanide–CeO_2_NPs SIA–CL system.

Ten standard solutions were used to test the SIA-CL linearity. Using the least square statistical analyses, the calculated regression equations were Y = 1.2786C + 17701, (r = 0.9990), Y = 34.802C +2121.8, (r = 0.9989), and Y = 2.3567C + 1759.1, (r = 0.9994) for ferulic acid, sinapic acid, and *p*-coumaric acid content, respectively. The concentration ranges of 20,000–200,000 ng mL^−1^ (ferulic acid), 1000–50,000 ng mL^−1^ (*p*-coumaric acid), and 50–4500 ng mL^−1^ (sinapic acid) were covered by the fitted calibration curve under optimum conditions and the linear regression equations (Figure 8).

#### 3.4.3. Limit of Detection (LOD) and Limit of Quantification (LOQ)

The lower limit of detection was used to assess the investigated compounds using the signal-to-noise ratio. The used equation, S/N = 2, means that the CL signal of the detected compound concentration is twofold that of the blank. The documented signals displayed great sensitivity, having a lower LOD of 17.500, 47.9, and 910 ng mL^−1^ for ferulic acid, sinapic, and *p*-coumaric acid, respectively. Moreover, a signal-to-noise ratio equal to 10 was used to assess the quantification limit, which was found to be 20,000, 50, and 1000 ng mL^−1^ for ferulic acid, sinapic, and *p*-coumaric acid, respectively.

#### 3.4.4. Accuracy, Precision, and Robustness

The mean percentage of recoveries was used to calculate accuracy. High accuracy was indicated by a calculated recovery percentage of 99.89 ± 1.2, 99.56 ± 0.9, and 99.18 ± 1.4% for ferulic acid, sinapic, and *p*-coumaric acid, respectively. The system precision was evaluated by employing intra-day and inter-day assays (Table 2). The mean relative standard deviation (% RSD) calculated from the test was 1.1%, 0.9%, and 1.0% for the intra-day assay, and 1.1%, 1.0%, and 1.1% for the inter-day assay, for ferulic acid, sinapic, and *p*-coumaric acid, respectively. It is well established that good precision is indicated by % RSD values less than 2% [2]. Moreover, several factors such as flow rate, aspiration rate and reagent and sample volume (100 ± 10 mL s^−1^, 30 ± 5 mL, and 60 ± 5 mL, respectively) were changed to affirm the technique robustness. The obtained results showed no significant differences (*p* ≤ 0.05) between the sample results and those documented using compound standards.

### 3.5. Antiradical Capacity

When radicals interact with oxygen, an oxidation chain reaction occurs, prolonging the oxidation damage. To block the damage expansion, antioxidants play a protective role based on quenching and neutralizing these radicals [28]. The antioxidant potential of low concentrations of active molecules is commonly assessed using the sensitivity of the DPPH• assay. The effect of various concentrations of MSE and CeO_2_NPs on DPPH• free scavenging capacity is presented in Table 3. The results demonstrated an increasing strong free scavenging capacity by both MSE and biogenic CeO_2_NPs in a dose-dependent manner. The capacity of the biogenic CeO_2_NPs was significantly superior to that of MSE in all concentrations. Elevating the concentration from 25 to 100 ug mL^−1^ increased the scavenging potential of the biogenic CeO_2_NPs by approximately 15% (from 60.21 to 75.11%).

Regarding the ABTS assay, potassium persulphate oxidates ABTS resulting, in the radical monocation of 2,2′-azino-bis(3-ethylbenzothiazoline-6-sulfonic acid) (ABTS• +). The capacity of different concentrations of MSE and biogenic CeO_2_NPs to reduce ABTS into ABTS^+^ ions was investigated. A similar trend was observed in this assay. Increasing the concentrations to 100 μg mL^−1^ of the CeO_2_NPs elevated the scavenging percentage from approximately 56 to 77% (Table 3).

The strong inhibitory outcome displayed by the biogenic CeO_2_NPs may be due to the synergistic effect of the antioxidants existing in the MSE and adsorbed onto the nanoceria surfaces. Since the nanomaterials undergo a significant reduction in size, they create an increasing total surface area, consequently exposing large numbers of active sites. These sites can function as catalytic centers for reduction reactions. The presented efficacy results, which were similar to those of the ascorbic acid capacity, indicate that MSE can be a powerful biological contributor of hydrogen-donating phenolics and flavonoids accountable for antioxidant capacity. Additionally, the intrinsic capabilities of the biogenic CeO_2_NPs with tunable physical and chemical parameters affected their surface relevant to the Ce^3+^/Ce^4+^ ratio, thus revealing antioxidant activities.

### 3.6. Levels of Ferulic, Sinapic, and p-Coumaric Acids

The developed SIA-CL technique was employed to determine the three phenolic compounds extracted from yellow maize kernels. Among the investigated compounds, ferulic acid was the predominant type in the kernel [29], with an amount of 1636 ± 2.61 ug/g_dw_. Similar levels of ferulic acid were presented by [30] in pigmented maize. Ferulic acid level was significantly higher than *p*-coumaric acid (206 ± 1.12 ug/g_dw_), the second highest phenolic compound, and sinapic acid (123 ± 2.15 ug/g_dw_). These findings were within the range reported by the findings of [31] using HPLC-MS/MS analysis. They reported that the highest levels of ferulic acid (1578- 1977 ug/g_dw_), *p*-coumaric acid (204–278 ug/g_dw_) and sinapic acid (117–151 ug/g_dw_) were found in yellow corn followed by barley, wheat, and then oats. In addition, a study presented by [32] using HPLC, reported a wider range of ferulic acid (1603–1838 ug/g_dw_) and *p*-coumaric acid (188–329 ug/g_dw_) in different maize accessions. The width of these ranges may be due to varietal differences, and environmental and growing conditions, in addition to extraction methods, which may affect the presence and distribution of the phenolic acid content.

Different studies have confirmed that the chemical components and pharmacological properties of maize silk have excellent reducibility characteristics and can act as reducing agents in the biogenic nanoparticle synthesizing process [33]. In this study, the results confirmed the presence of various functional groups that may be related to the phytochemicals in maize silk extract.

Variations in ROS not only influence the stability and functionality of biological molecules, but also change the redox mediated signaling of the cell. The biogenic CeO_2_NPs exhibit several unique characteristics that enhance their availability as a potential therapeutic tool in an array of biological applications that demonstrate oxidative stress. The surface Ce^3 +^/Ce^4+^ valency switch provides the ROS scavenging capability that allows CeO_2_NPs to act as a biological antioxidant. Thus, when interacting with various ROS species, its redox potential is distinctively positioned to interface with ROS and decrease the influence of radicals [34].

Generally, nanoparticles influence the luminol–ferricyanide CL reaction in a particle size-dependent manner [35]. Very small CeO_2_NPs (less than 5 nm in size) can negatively influence the CL reaction of luminol and ferricyanide as a result of the high surface energy produced from the minute particles, consequently increasing the redox activity. By comparison, a particle size in the range of 10–100 nm augments the CL reaction because of the catalytic enrichment and oxidation state changes involved in the electron transfer process. Under the condition of this study, the significant sharp CL signal obtained using the biogenic CeO_2_NPs suggests the active surface facilitation of oxidating luminol and the formation of superoxide intermediate radicals that enriched the CL signal [36]. Thus, the ultrasensitivity of the developed scheme supported the applicability of its usage in assessing and detecting different phenolic compounds in biological samples.

However, more investigations are needed to test the antifungal and antibacterial capabilities, the anticancer effects using human cancer cell lines, and the antidiabetic properties, in addition to the impact of these biogenic synthesized CeO_2_NPs on maize yield, gemination, growth, and nutritional parameters when used for seed nano-priming.

## 4. Conclusions

The current study elucidated the possibility of using maize silk, due to its strong bioactivities, to provide a green, modest, and easy platform for the biosynthesis of CeO_2_NPs. Quantitative determination of ferulic, sinapic, and *p*-coumaric acids was carried out using an eco-friendly, cost-effective, ultrasensitive, and nanoceria-enhanced sequential injection-chemiluminescence (SIA-CL) system. The suggested system showed an outstanding sensitivity and dependability in quantifying the three phenolic compounds at the nanoscale level. The antioxidant activity of maize silk and the biogenic CeO_2_NPs were tested and exhibited a strong scavenging potential. The intrinsic capabilities of biogenic CeO_2_NPs in enhancing the developed system revealed its potential role in detecting phenolic compounds with great sensitivity.

## Figures and Tables

**Table 1:** Tolerable levels of possible interfering species in the determination of phenolic compounds (ferulic acid, sinapic acid, *p*-coumaric acid) of maize kernels.

Interferents Species	Tolerable Level (ng mL^−1^)
Na^+^, K^+^, Fe^3+^, Mn^3+^, Mg^2+^, Ca^2+^, Cu^2+^ and Zn^2+^	1,200,000
Lysine and tryptophan	600,000
Starch, sucrose, glucose, and fructose	100,000
Vitamin E, thiamine (vitamin B1), riboflavin (vitamin B2), niacin (vitamin B3), and ascorbic acid	800,000

**Table 2:** Intra-day and inter-day assays for CeO_2_NPs SIA-CL system determination of ferulic, sinapic, and *p*-coumaric acids in maize kernels.

Sample	Precision % RSD	
Ferulic acid conc. (ng mL^−1^)	Intra-day	Inter-day
20,000	1.2	1.1
100,000	1.4	0.9
200,000	0.8	1.3
Sinapic acid conc. (ng mL^−1^)	Intra-day	Inter-day
50	0.5	0.9
1500	1.5	1.2
4000	0.9	1.1
*p*-Coumaric acid conc. (ng mL^−1^)	Intra-day	Inter-day
1000	1.4	1.2
25,000	1.1	1.3
50,000	0.7	0.8

**Table 3:** DPPH• and ABTS scavenging activities for different concentrations of MSE and biogenic CeO_2_NPs, and ascorbic acid as the control.

	DPPH Radical Scavenging Activity		ABTS Radical Scavenging Activity	
Samples	Concentration (ug mL^−1^)	Scavenging (%)	Concentration (ug mL^−1^)	Scavenging (%)
MSE	25	35.69 ± 0.53	25	37.21 ± 0.13
	50	42.84 ± 0.27	50	47.42 ± 0.26
	100	55.89 ± 0.22	100	52.44 ± 0.45
CeO_2_NPs	25	60.21 ± 0.55	25	55.81 ± 0.49
	50	66.90 ± 0.32	50	69.95 ± 0.19
	100	75.11 ± 0.14	100	75.70 ± 0.76
Ascorbic Acid	25	62.43 ± 0.42	25	59.22 ± 0.32
	50	71.87 ± 0.61	50	71.17 ± 0.89
	100	79.12 ± 0.31	100	77.56 ± 0.61

## Data Availability

The data used to support the findings of this study are included within the article.
